# Editorial: Microbial biodiversity and bioprospecting in polar ecosystems in the genomics era

**DOI:** 10.3389/fmicb.2024.1504105

**Published:** 2024-10-23

**Authors:** Carmen Rizzo, Syed Gulam Dastager, Hilal Ay

**Affiliations:** ^1^Department of Sustainable Marine Biotechnology, Anton Dohrn Zoological Station Naples, Naples, Italy; ^2^NCIM Resource Center, Biochemical Sciences Division, National Chemical Laboratory (CSIR), Pune, India; ^3^Department of Molecular Biology and Genetics, Faculty of Arts and Science, Yildiz Technical University, Istanbul, Türkiye

**Keywords:** genomics, Antarctica, Arctic, biodiversity, biotechnology, genome mining, metagenome-assembled genome (MAG)

Microorganisms are the most diverse and abundant organisms essential for biogeochemical cycles and the functioning of ecosystems. They have also been considered the most profitable sources of bioactive natural products for over a century. Therefore, microbial diversity is crucial for the sustainability of ecosystem function and biotechnological applications of microbial natural resources, particularly for health-related advances. In this regard, polar and subpolar habitats, including microbial symbioses, are paramount for maintaining biodiversity and community composition, as global climate change can dramatically affect these extreme and unique environments (Bhat et al., 2022). In recent decades, the emergence of genomic technologies has provided insights into understanding the diversity, function, adaptation and evolution of microorganisms and microbial communities in diverse global environments (Ramasamy et al., 2023). Genome-resolved metagenomic studies have significantly expanded the family tree of life by providing a clear representation of the phylogenetic diversity of microorganisms (Royo-Llonch et al., 2021; Pessi et al., 2023; Greco et al., 2024). Therefore, more comprehensive studies of microbial biodiversity in polar habitats could help model the impacts of global climate change on these vulnerable ecosystems and provide new sources for bioprospecting.

Due to the extreme physicochemical conditions of polar environments, bioactive secondary metabolites produced by polar microorganisms can exhibit enormous scaffold diversity and structural complexity (De Pascale et al., 2012). In addition, cold-active genes from Arctic and Antarctic microorganisms are considered valuable sources of antifreeze proteins, extracellular polymeric substances and polyunsaturated fatty acids with potential applications in medicine, agriculture, food, and textile industries (Ramasamy et al., 2023). Although several natural products with bioactivity and cold-active enzymes have been isolated from polar and subpolar environments (Prasad et al., 2014), there is an urgent need to uncover the microbial biodiversity and bioprospecting potential of these environments by using culture-dependent and culture-independent genomics approaches, including the construction of metagenomic assembled genomes. Understanding the microbial and chemical diversity of Arctic and Antarctic habitats also offers the opportunity to relate this diversity to ecosystem and evolutionary parameters.

The articles published in the “Research Topic” collection addressed various aspects of polar microbial ecology and bioprospecting, highlighting the importance of exploring extremophilic microorganisms from cryosphere environments as untapped reservoirs of chemical diversity based on their specialized adaptive strategies necessary for survival in harsh environmental conditions. The study provided by Jaarsma et al. aimed to investigate the bacterial biosynthetic potential of glaciers and ice sheets, which are considered microbially controlled biomes and are generally dominated by *Pseudomonadota, Actinomycetota* and *Bacteroidota*. A genome mining approach was applied to whole genomes of 28 bacterial isolates and 133 high-quality metagenome-assembled genomes (MAGs) obtained from supraglacial habitats on the Greenland ice sheet (ice sheet surface, cryoconite, biofilm, and snow). The study allowed us to obtain a comprehensive set of predicted biosynthetic gene clusters (BGCs), with the mean number for the isolated genomes being higher than for the MAGs, demonstrating a strong adaptive potential to cope with extreme environments. In fact, the gene clusters discovered were mainly related to the production of antimicrobial agents, carotenoid pigments, siderophores, and osmoprotectants. Furthermore, the results showed that most identified BGCs have unknown functions, suggesting significant and yet unexplored potential for biological discovery.

Modern methodologies based on omics technology could be used for more microbial ecology-oriented investigations, such as exploring underexplored areas and studying the function of communities in ecosystem dynamics. Notably, contemporary sequencing and post-run analysis techniques apply to various targets, allowing observations to be made on both prokaryotes and eukaryotes. Thøgersen et al. investigated the bacterial and eukaryotic diversity of ikaite columns in the Ikka Fjord in Greenland using 16S and 18S *rRNA* gene amplicons and metagenomic sequencing. In this example, the MAGs were examined for the presence of genes involved in nitrogen, phosphorous, and sulfur cycling, as well as the production of carbohydrate-utilizing enzymes (CAZymes). Bacteria, archaea, and eukaryotes (including microalgae, protists, fungi, and tiny animals) represented by 12, 7, and 27 phyla, respectively, were found across the columns. A significant presence of sulfur-cycling genes, together with prior observations of sulfur in the water inside the ikaite columns (Buchardt et al., 2001), indicates that sulfur plays an important role in the metabolic strategies of microorganisms within the ikaite columns. In terms of biotechnology, the study highlighted the identification of carbohydrate-active enzymes that support the potential and ecological functions of microbial communities associated with these unique mineral formations, as well as the diversity of carbohydrate-metabolizing enzymatic activities.

An ecological approach was also provided by Bruhn et al., whose study describes the temporal changes in protist community composition from winter to spring in the Arctic marine ecosystem. The authors described a different seasonal taxonomic composition, with a protist community consisting of parasites, heterotrophs, and mixotrophs during winter, probably adapted to low light availability conditions. The transitional period was characterized by a more flexible trophic community, with an abundance of mixotrophs, as well as a clear shift toward a spring bloom before the sea ice receded, in accordance with the hypothesis of various factors involved in the phytoplankton spring bloom, including light intensity, spectral composition, and day-length, oceanographic parameters.

Buschi et al. provide new insights into the knowledge of the microbiomes associated with Antarctic invertebrates, expanding information on diversity, functions, and origin of bacterial taxa belonging to the microbiomes. Although a bacterial core was observed in the *Odontaster validus* specimens investigated (mostly represented by the family *Rhodobacteraceae*), the richness and taxonomic composition of the microbiomes significantly changed among different Antarctic sectors and within a single area (e.g., Ross Sea). This suggests that besides the geographic sector, other environmental and/or biological factors may influence the microbiome composition of these Antarctic invertebrates. Members belonging to the bacterial core (including *Propionibacteriaceae* and *Bacillaceae* in addition to *Rhodobacteraceae*) may play a fundamental role in the sea stars' wellbeing, potentially establishing commensalism and symbiotic relationships with their hosts and contributing to the metabolic pathways of a wide array of inorganic and organic compounds. Since bacteria belonging to the *Roseobacter* genus were found not only in all specimens of *O. validus* but also in their surrounding sediments, this indicates a selection mechanism of the host from the environment to acquire these key holobiont members.

de Lemos et al. provided an example of *Paenibacillus antarcticus* strain IPAC21 isolated from Antarctic soil, a producer of bioemulsifier. A whole genome study reveals the genes and metabolic pathways related to bioemulsifier production. It shows the presence of genes associated with adaptation to environmental stress conditions such as cold response, membrane fluidity mechanisms, DNA repair, oxidative and osmotic stress, chaperones and transport of compatible solutes. Genes for the biosynthesis of the bioemulsifier levan are described and compared to those of closely related *Paenibacillus* species. All the data contributed to a better knowledge of this psychrotolerant strain isolated from Antarctic soil, showing potential biotechnological applications in the petroleum industry.

## References

[B1] Bhat B. A. Tariq L. Mir R. A. Majeed I. Bandh M. M. (2022). “The vulnerability of microbial ecosystems in a challenging climate,” in Climate Change and Microbes (Burlington: Apple Academic Press), 51–79. 10.1201/9781003189725-3

[B2] Buchardt B. Israelson C. Seaman P. Stockmann G. (2001). Ikaite tufa towers in Ikka Fjord, Southwest Greenland: their formation by mixing of seawater and alkaline spring water. J. Sediment. Res. 71, 176–189. 10.1306/042800710176

[B3] De Pascale D. De Santi C. Fu J. Landfald B. (2012). The microbial diversity of Polar environments is a fertile ground for bioprospecting. Mar. Genomics 8, 15–22. 10.1016/j.margen.2012.04.004 23199876

[B4] Greco C. Andersen D. T. Yallop M. L. Barker G. Jungblut A. D. (2024). Genome-resolved metagenomics reveals diverse taxa and metabolic complexity in Antarctic lake microbial structures. Environ. Microbiol. 26:e16663. 10.1111/1462-2920.16663 38881221

[B5] Pessi I. S. Popin R. V. Durieu B. Lara Y. Tytgat B. Savaglia V.. (2023). Novel diversity of polar Cyanobacteria revealed by genome-resolved metagenomics. Microbial. Genomics 9:001056. 10.1099/mgen.0.001056 37417735 PMC10438808

[B6] Prasad S. Manasa P. Buddhi S. Tirunagari P. Begum Z. Rajan S.. (2014). Diversity and bioprospective potential (cold-active enzymes) of cultivable marine bacteria from the subarctic glacial fjord, Kongsfjorden. Curr. Microbiol. 68, 233–238. 10.1007/s00284-013-0467-6 24121613

[B7] Ramasamy K. P. Mahawar L. Rajasabapathy R. Rajeshwari K. Miceli C. Pucciarelli S. (2023). Comprehensive insights on environmental adaptation strategies in Antarctic bacteria and biotechnological applications of cold adapted molecules. Front. Microbiol. 14:1197797. 10.3389/fmicb.2023.1197797 37396361 PMC10312091

[B8] Royo-Llonch M. Sánchez P. Ruiz-González C. Salazar G. Pedrós-Alió C. Sebastián M.. (2021). Compendium of 530 metagenome-assembled bacterial and archaeal genomes from the polar Arctic Ocean. Nat. Microbiol. 6, 1561–1574. 10.1038/s41564-021-00979-9 34782724

